# Does the orientation of syndesmosis fixative device affect the immediate reduction of the distal tibiofibular joint?

**DOI:** 10.1007/s00402-021-04073-x

**Published:** 2021-08-07

**Authors:** Robert Hennings, Ulrich J. Spiegl, Carolin Fuchs, Pierre Hepp, Johannes K. M. Fakler, Annette B. Ahrberg

**Affiliations:** Department of Orthopaedics, Traumatology and Plastic Surgery, University of Leipzig, Liebigstr. 20, 04103 Leipzig, Saxony Germany

**Keywords:** Syndesmosis, Tibiofibular, Syndesmotic screw, Suture button

## Abstract

**Introduction:**

Incongruent stabilization of the distal tibiofibular joint (syndesmosis) results in poorer long-term outcome in malleolar fractures. The aim was to analyze whether the orientation of the syndesmotic stabilization would affect the immediate reduction imaged in computed tomography (CT).

**Materials and methods:**

The syndesmotic congruity in 114 ankle fractures with stabilization of the syndesmosis were retrospectively analyzed in the post-operative bilateral CT scans. The incisura device angle (IDA) was defined and correlated with the side-to-side difference of Leporjärvi clear-space (ΔLCS), anterior tibiofibular distance (ΔantTFD) and Nault talar dome angle (ΔNTDA) regardless of the stabilization technique and separately for suture button system and syndesmotic screw. Asymmetric reduction was defined as ΔLCS > 2 mm and |ΔantTFD|> 2 mm.

**Results:**

Regardless of the stabilization technique, no correlation between the IDA and the ΔLCS (*r* = 0.069), the ΔantTFD (*r* = 0.019) nor the ΔNTDA (*r* = 0.177) could be observed. There were no differences between suture button system and syndesmotic screw. Asymmetrical reduction was detected in 46% of the cases, while sagittal asymmetry was most common. No association was found between the orientation of stabilization device and occurrence of asymmetrical reduction (*p* > 0.05). The results of suture button system and syndesmotic screw were comparable in this respect (*p* > 0.05).

**Conclusion:**

Poor correlation between the orientation of the stabilization device and the immediate post-operative congruity of the syndesmosis could be shown. In contrast to current literature, this study did not show difference of suture button system over syndesmotic screw in this regard. Careful adjustment of the fibula in anteroposterior orientation should be given special attention.

## Introduction

Ankle fractures are one of the most common fractures [1]. Up to 39% are associated with an instability of the distal tibiofibular joint (syndesmosis) requiring stabilization [2]. An anatomical reduction of the fracture components as well as the syndesmosis is the prerequisite for a good post-operative outcome and the prevention of post-traumatic arthrosis [3–6]. If a syndesmotic lesion is detected, reduction and stabilization of the fibula position in the tibia incisura is indicated. With the static syndesmosis screw (SYS) and the flexible Suture Button System (SBS), equivalent stabilization methods are available for this purpose [8–10]. The general recommendation is to perform stabilization in an ascending orientation from posterolateral to anteromedial in the transversal plain at an angle of 20–30° with the foot in neutral position [7]. In contrast, two recent studies examining the orientation of the stabilization in relation to individual anatomy recommend a reduced ascending orientation of 18.8° [8, 9]. Furthermore, several computed tomographic (CT) analyses revealed significant interindividual anatomic differences of the syndesmosis [10–12]. By identifying risk factors and improving surgical technique, syndesmotic malreduction rates were reduced from 52% to around 20% with superiority of SBS [13–16]. However, the studies available have shown that stabilization of the syndesmosis is still a critical surgical step and syndesmotic asymmetry is associated with worse post-operative results [5, 14, 17]. With all studies already conducted in this field, there is still lack of evidence about the relationship between the rate of syndesmotic incongruity and the orientation of the stabilization device [9, 18, 19]. Therefore, the aim of the study was to evaluate the influence of the orientation of the syndesmosis stabilization device on the immediate post-operative syndesmotic congruity imaged in computed tomography. The hypotheses of the study were that the orientation of the stabilization device has no influence on the immediate reduction result and both stabilization methods (SYS, SBS) are comparable in this respect.

## Patients and methods

Approval of the local institutional review board was given beforehand (AZ 488/19-ek) and the study was conducted in accordance with the Declaration of Helsinki and the guidelines for Good Clinical Practice.

Consecutive adult patients who received surgical stabilization of the syndesmosis between 01/2010 and 12/2019 were included in this retrospective study. All fractures were classified in accordance with the Arbeitsgruppe für Osteosynthesefragen (AO) classification [7, 20]. Ankle fracture types 44-B and 44-C (*N* = 183) were identified and stored pseudonymized in electronic data base using SPSS (version 24, Chicago, IL, USA) [7, 20]. Further inclusion parameters were post-operative bilateral CT control, anatomical stabilization of the fracture components, and an unsuspicious contralateral ankle without a history of ankle fractures in the past or other ankle deformities (*N* = 114). Exclusion criteria were degenerative alterations of the uninjured ankle and insufficient CT scans that did not include the fixation device. Additionally, patients with persistent bone step > 2 mm with non-anatomic reduction of the fractures were excluded, based on the finding that fibular shortening of > 2 mm after osteosynthesis represents an isolated risk factor for malalignment of the syndesmosis [21].

### Operative management

All patients were treated according to the recommendations of the AO [11]. If not evident from the preoperative imaging, syndesmotic instability was evaluated under standard fluoroscopy (lateral and mortise view) following fracture stabilization using the hook test while the ankle was placed in neutral dorsiflexed position [7, 22–25]. After detecting the instability, reduction of the syndesmosis and stabilization was performed under direct visualization and fluoroscopy with a quadricortical, fully threaded 3.5 mm syndesmotic screw (DePuy-Synthes) or a suture button device (TightRope®, Arthrex, Naples, FL, USA) [22, 26]. All operations were performed by experienced specialists in ankle surgery of a trauma level I center. The surgeons decided which stabilization device to use based on their intraoperative assessment of bone quality, their experience with the devices and preference. In suspicion of osteoporosis, syndesmotic screws were used. Reduction and fixation were controlled by fluoroscopy intraoperatively.

### Groups

Based on the stabilization procedure, two study groups were defined. The SYS group included patients who received a stabilization with a syndesmotic screw (*N* = 42; 37%) and the SBS group, who were stabilized using a suture button system (*N* = 72; 63%).

### CT scan image analysis

**Fig. 1:**
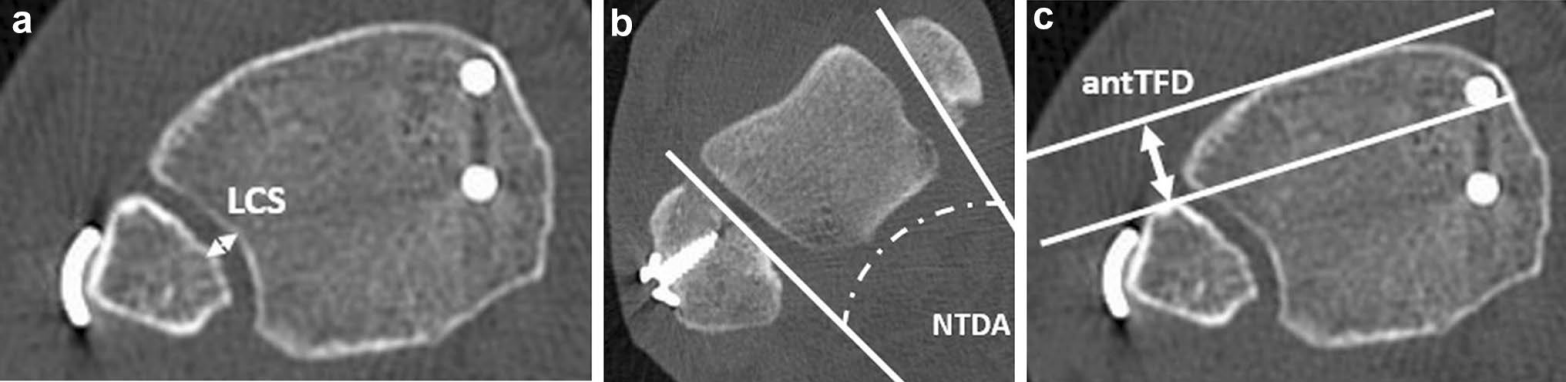
Transversal plane of computer tomography 10 mm above the ankle joint demonstrate the measurement of Leporjärvi Clear Space (LCS; 2a), Nault talar dome angle (NTDA; 2b) and anterior tibiofibular distance (antTFD; 2c) at the injured ankle joint [28–30]

**Fig. 2:**
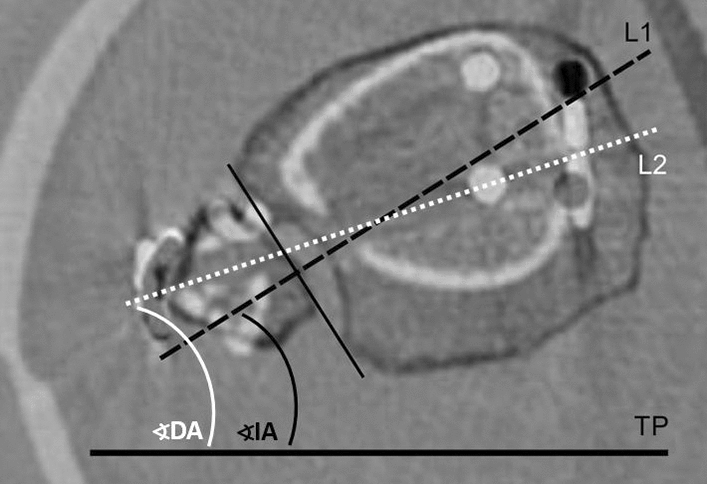
Illustration of the transversal plane of computer tomography 10 mm above the plafond (black contours) and the plane at the level of the tibiofibular stabilization (white contours). L1 (black dashed line) = perpendicular line crossing the midpoint between the anterior tubercle and posterior tubercle of the tibial incisura; L2 (white dotted line) = tangent along the axis of the fixation device; *TP* transversal plane; angle between L1 and TP = Incisura Angel (IA); angle between L2 and TP = Device Angel (DA)

All non-weightbearing CT scans were obtained during the in-patient period without administration of intravenous contrast medium as part of the standard care to assess syndesmotic reduction. Patients were positioned supine and feet first with the ankle in neutral position with both ankles in the same scanning field. Images were acquired using a multidetector CT scanner (iCT 256, Philips, Netherlands) Routine scan parameters included a tube current of 150 mA, a tube voltage of 100 kV with a collimation of 64 × 0.625 mm. Pitch was 0.329 with a rotation time of 0.5 s. Multiplanar reformations were reconstructed in slice thickness of 0.67–2 mm in axial, sagittal and coronal orientation. Syndesmotic reduction was assessed 10 mm proximal of the ankle joint using validated landmark-based techniques according to Schon et al. [27, 28]. Measurements were made using SieNet MagicWeb (Siemens AG, Medical Solutions, Germany) by one experienced orthopedic and trauma surgeon who was trained in the measurement methods. The Leporjärvi Clear Space (LCS) was used to analyze the medial–lateral translation. The Nault talar dome angle (NTDA) was measured to evaluated the external rotation of the fibula (Fig. 1a and b) [27, 29, 30]. The anterior–posterior translation was assessed to define the anterior tibiofibular distance according to Ahrberg et al.(antTFD; Fig. 1c) [28]. These parameters were selected due to their high intra-observer and inter-observer reliability in evaluating side differences as demonstrated in the literature and were assessed for both sides [27, 28]. The side-to-side differences between injured and uninjured sides were calculated and defined as ΔLCS, ΔNTDA and ΔantTFD. Positive ΔLCS represented widening of the syndesmosis. Positive ΔantTFD was defined as a posterior translation of the fibula in relation to the tibia at injured side, whereas positive ΔNTDA represented an increased external rotation of the fibula of the injured side. ΔLCS > 2 mm and |ΔantTFD| of more than 2.0 mm were considered as asymmetrical syndesmotic reduction in accordance to literature [5, 15]. The orientation of the syndesmotic fixation device and tibial incisura (SBS, SYS) was assessed as shown in Fig. 2, adapted from the technique used by Park et al. [8]. The measurement was performed 10 mm above the plafond in an axial plane. A tangent crossing the midpoint of the connection between the anterior tubercle to posterior tubercle of the tibial was defined as reference line 1 (line 1; L1) [12, 31]. The angle between L1 and the transverse plane (TP) was defined as Incisura Angle (IA, Fig. 2). To determine the orientation of the fixation device, the angle between the tangent along the axis of the fixation device (line 2; L2) and the TP was measured (Device Angle, DA, Fig. 2). Subtraction of the IA from the DA was defined as the incisura device angle (IDA).

### Statistics

Statistical analysis was performed with SPSS software (Version 25, IBM, Chicago, IL, USA). The Student’s *t*-test or Mann–Whitney *U*-test was used to compare continuous variables between the study groups. Categorical variables were compared using Pearson’s Chi-square test or Fisher’s exact test. Pearson correlation coefficients (*r*) were used for correlation analysis and were interpreted as poor ( *r* < 0.4), acceptable (0.4 < *r* < 0.59), good (0.6 < *r* < 0.74), or excellent (> *r* > 0.74) [32]. Differences between means with 95% confidence intervals (95% CI) were calculated. Two-tailed *p*-values are presented. A *p*-value *p* < 0.05 was considered to be significant. After applying the inclusion and exclusion criteria, the maximum sample size in this retrospective study was 114 patients. Power analysis showed that the minimum correlation coefficient considered significant was *r* = 0.328 for *N* = 114 and for the subgroups SBS (72 patients) *r* = 0.406 and SYS (42 patients) *r* = 0.516.

## Results

The mean age of the patients was 44.5 years (18–85 years, SD 16.6). Men were younger than women (*p* = 0.001; Table 1). In total, 72 patients (63%) were stabilized with a suture button system (SBS group). The SBS group (*N* = 72) was younger compared to the SYS group (SYS group; *N* = 42; *p* = 0.010, Table 1).

**Table 1:** Patients characteristics, fracture pattern and outcome for the total patient cohort and comparison of both stabilization procedures

	All patients *N* = 114		*p*-value
	Women; N = 52	Men; N = 62	
Mean age in year (SD)	50.2 (18.1)	39.6 (13.5)	**0.001**^**a**^
IDA in	− 10 (11; − 35 to 16)		
ΔLCS in mm	0.5 (1.6; − 4.6 to 5.5)		
ΔantTFD in mm	0,5 (2,8; − 7.7 to 9.0)		
ΔNTDA in	3.7 (7.8¸− 30 to 19		
Correlation regardless types of stabilization (*r*)			
IDA and ΔLCS	0.069		
IDA and ΔantTFD	0.019		
IDA and ΔNTDA	0.177		

	Stabilization		
	SBS group; *N* = 72	SYS group; *N* = 42	
age in year	41.4 (15.2; 18–80)	49.6 (17.7; 19–85)	**0.010**^**a**^
female: male	34:38:00	18:24	0.652^b^
Anatomy and osteosyntheses of the fractures, N			
Isolated fibula	25	13	
Fibula and medial mall	12	7	
Fibula and posterior mall.^c^	17	5	
Fibula and posterior mall.^d^	3	3	
Fibula, medial and posterior mall.^c^	11	9	
Trimalleolar fracture^d^	4	5	
IDA in°	− 10 (11; − 35 to 14)	− 11 (13; − 32 to 14)	0.893^a^
ΔLCS in mm	0.6 (1.5)	0.4 (1.8)	0.580^a^
ΔantTFD in mm	0.9 (2.6)	− 0.3 (2.9)	**0.020**^a^
ΔNTDA in °	4 (8)	3 (6)	0.415^a^
Correlation (*r*)			
IDA and ΔLCS	0.245	− 0.143	
IDA and ΔantTFD	− 0.086	0.152	
IDA and ΔNTDA	0.22	0.104	

Bold *p* values highlights significant differences

All data are presented as mean (SD; range). *Δ* side-to-side difference, *IDA * Incisura device angle, *LCS* Leporjärvi Clear Space, antTFD = anterior tibiofibular distance, *NTDA* Nault talar dome angle, *SBS* suture button system, *SYS* syndesmostic screw

^a^Student`s *t*-test

^b^*χ*^2^-test; mall. = malleolus

^c^Posterior malleolus no fixation

^d^Posterior malleolus fixed

Regardless of the fixation device, average IDA was − 10° (SD 11°; range − 35° to 16°). There was no difference between stabilization systems (*p* = 0.893; Table 2). Regardless of the stabilization device, poor correlation between IDA and ΔLCS (*r* = 0.069), ΔantTFD (*r* = 0.019) and the ΔNTDA (*r* = 0.177) could be observed (Table 1).

**Table 2:** Overview of the reduction outcome and its relation with the parameters of interest. All data are presented as mean (SD)

	Reduction outcome		*p*-value
	Anatomical reduction	Asymmetric reduction^*^	
ΔantTFD N	62	38	
IDA in °	− 10 (11)	− 12 (11)	0.221^a^
Correlation (*r*) of			
IDA and ΔantTFD	0.024	0.08	
ΔLCS N	62	11	
IDA in °	− 10 (11)	− 6 (12)	0.282^a^
Correlation (*r*) of			
IDA and ΔLCS	0.024	− 0.363	

	Ant. asymmetry	Post. asymmetry	
Malreduced acc. ΔantTFD (*N* = 38)	10	28	
ΔantTFD in mm	− 4.1 (2.1)	3.7 (2.0)	**0.013**
IDA in °	− 16 (13)	− 11 (10)	0.205^b^
LCS in mm	3.2 (1.6)	3.9 (1.4)	0.191^b^
ΔLCS in mm	− 0.1 (1.9)	0.5 (1.3)	0.310^b^
ΔNTDA in °	3 (7))	6 (6)	0.299^b^

Bold *p* value highlights significant differences

*Unidirectional malreduction; *ant.* = anterior, *post.* Posterior, *IDA* incisura device angle, *LCS* Leporjärvi Clear Space, *antTFD* anterior tibiofibular distance, *NTDA* Nault talar dome angle

^a^student *t*-test

^b^Mann–Whitney *U* Test

In the separate analysis of the SBS group and SYS group, poor correlation was found between the IDA and ΔLCS, ΔantTFD or the ΔNTDA (Table 1). The mean ΔLCS and ΔNTDA were comparable between the groups (*p* > 0.05; Table 1). AntTFD differed between both groups, with SBS positive in mean and SYS negative in mean (*p* = 0.020; Table 1).

An isolated syndesmotic asymmetry according to ΔantTFD was seen in 38 patients (33%), and in 11 patients (10%) according to ΔLCS. A combined syndesmotic asymmetry (ΔantTFD and ΔLCS) was visible in three patients (3%). Thus, in a total of 52 patients (46%) asymmetric syndesmosis was shown. Posterior translation was more common in the SBS group compared to the SYS group (SBS 22 vs SYS 9; *p* ≤ 0.003; *r* = 0,389). The mean IDA of patients defined as anatomically reduced (-10°) and those with a syndesmotic asymmetry was comparable for both ΔantTFD (− 12°; *p* = 0.221, and ΔLCS (− 6°; *p* = 0.282, Table 2). Furthermore, there was no difference assessing IDA between patients with anterior or posterior asymmetrical tibiofibular position (*p* = 0.205; Table 2). The mean values of absolute LCS as well as ΔLCS of patients with anterior asymmetry were comparable to those of posterior asymmetrical tibiofibular position (*p* > 0.05; Table 2). Poor correlation was evident between patients evaluated as having anterior, respectively, posterior tibiofibular asymmetry and IDA, ΔLCS or ΔNTDA (− 0.200 < *r* < 0.200).

## Discussion

The hypotheses of the study were that the orientation of the stabilization device (SBS, SYS) does not affect the immediate reduction result and both stabilization methods are comparable in this respect.

The data have demonstrated a wide range of the orientation of the stabilization devices. However, the orientation does not influence the immediate congruity and the rate of asymmetrical syndesmotic reduction in side-to-side consideration analyzed in CT. Asymmetry in sagittal orientation was the most common failure of syndesmotic incongruity. This is comparable for both stabilization devices. Thus, the hypotheses have to be retained.

The AO recommends an oblique postero-fibular to antero-tibial angle of approximately 30° for screw trajectory in transversal plain with the foot in a neutral dorsiflexion position with 20° internal rotation [22, 33]. Park et al. have shown that adjustment to the second toe, which is perpendicular to the ground is reliable methods, whereby the ideal angle of the device should be 18.8° in transversal plain [8]. However, studies have shown a large interindividual variability in the anatomy of the distal syndesmosis, which could explain the large variance in the orientation of the stabilizing device despite standardized surgical technique [11, 17, 34–37].

Generally, there is limited evidence examining the relationship between the orientation of stabilization device and post-operative congruity of the syndesmosis. In contrast to our results, cadaver studies have demonstrated that the orientation of the stabilization device influences reduction result and varies depending on the entry point of the syndesmotic screw in the fibula [9]. Accordingly, a posterior orientation of syndesmotic fixation causes posterior translation and anteriorly oriented syndesmotic fixation causes anterior translation of the fibula with respect to the tibia [9]. Furthermore, Nimick et al. have found an anterior asymmetry of the syndesmosis in post-operative CT controls when the fixation was positioned anteriorly and a posterior asymmetry when the fixation was positioned more posteriorly. Based on their results, the authors recommended an orientation of stabilization device in the anterior third of the tibia in line with transmalleolar axis [18]. In the studies referred to above, the syndesmosis was reduced with reduction forceps before definitive fixation [9, 18]. There is no description whether asymmetry had been ruled out during this step. Syndesmotic asymmetry has been shown to be related to the position of the reduction forceps [9, 19]. A preliminary fixation with a deviation from the transmalleolar axis can provoke asymmetrical syndesmotic reduction [9, 19]. Cosgrove et al. have demonstrated that a clamp position laterally centered on the fibula and medially on the anterior third of the tibia assessed at the talar dome most often results in anatomical reduction [19].

Because of interindividual anatomic differences in syndesmotic width and morphologic variants, bilateral CT control is superior to conventional radiography [10, 11, 13, 28, 35, 37]. This has shown rates of syndesmotic malreduction between 0 and 42%, with various definitions [5, 10, 14, 14]. In contrast to the literature, we could not show a superiority of the SBS compared to the SYS in this respect [10, 14–16]. We explain our high rate of asymmetric syndesmosis reduction (46%) by the separate evaluation of sagittal translation (33%) and diastasis (10%). Comparable to the literature, asymmetry in sagittal plane was most common in this analyzes, with 33% of cases [38]. For the chosen cut-off value of more than 2 mm in side-to-side comparison for evaluation as syndesmotic asymmetry, an association with worse post-operative results has been shown and is commonly referred to as malreduction. [5, 15]. Therefore, it is important to identify any preventable and correctable cause for asymmetries. But in spite of several studies, little is evident about the relationship between the rate of syndesmotic asymmetry and the orientation of the stabilization [9, 18, 19]. Our results have shown no causation between the orientation of the stabilization device and the syndesmotic congruity, neither in SYS nor SBS. In the SBS, asymmetrically dorsal translational was more frequent than in the SYS with the same rate of syndesmotic malreduction in the sagittal plane. It is controversial whether there is some spontaneous reduction of an asymmetry in sagittal plane in the SBS after weight bearing or bracing, respectively, after removal of SYS which should be taken into account if revision is considered [16, 39]. Therefore, the definition of an isolated malreduction in sagittal plane is not conclusive and further research on these patients is needed.

Based on these results, we assume that post-operative syndesmotic asymmetry is not primarily caused by deviation of the stabilization device from the ideal trajectory. Rather, it is influenced by the quality of bony reduction of the tibial and fibular fractures as well as the restoration of the tibiofibular congruity prior to syndesmotic fixation.

Along with the retrospective study design, one limitation of this study is that the CT scans are performed without stress or weight bearing. However, intraoperative CT imaging is gaining in importance, which is also unloaded. It has been shown that 2D measurements cannot fully describe the three-dimensional relationships of the syndesmosis as it is possible by weightbearing cone-beam CT (WBCT) [40, 41]. Yet, Hamard et al. have shown that unloaded multidetector computed tomography is more capable of distinguishing pathologic syndesmoses than WBCT [42]. On the one hand, the upright position causes a widening of normal ankle size; on the other hand, the injured leg is not fully loaded due to pain [42]. In the fracture situations, non-weightbearing cone-beam CT may offer a low-radiation alternative to multidetector CT [40].

Another limitation is that clinical outcomes were not assessed. Regardless, the primary objective of this retrospective CT analysis was to evaluate the influence of the orientation of syndesmotic stabilization device on immediate post-operative radiological outcomes. However, there was a wide range of IDAs, so there is a risk that the study might have been underpowered.

Further studies are required to analyze the influence of the orientation of the stabilization device on the clinical outcome. This question arises particularly in the context of implantation of an SBS, which is left in place, in contrast to the SYS, which is usually removed.

In conclusion, contrary to the available literature, we did not observe any correlation between the orientation of the stabilization device and the immediate post-operative congruity of the syndesmosis visualized in CT. Special attention should be paid to the careful intraoperative adjustment of the fibula in sagittal orientation. Based on the results, we assume that the quality of the bony reduction and the restoration of the tibiofibular congruity before syndesmotic fixation influences the post-operative position more than the orientation of the stabilization device itself.

## Data Availability

The datasets used and/or analyzed during the current study are available from the corresponding author on reasonable request.
